# Bone-conduction threshold and air-bone gap may predict frequency-specific air-conduction threshold after tympanoplasty

**DOI:** 10.1371/journal.pone.0248421

**Published:** 2021-03-11

**Authors:** Ethan I. Huang, Yu-Chieh Wu, Hsiu-Mei Chuang, Tzu-Chi Huang

**Affiliations:** 1 Department of Otolaryngology, Chang Gung Memorial Hospital, Chiayi, Taiwan; 2 School of Medicine, Chang Gung University, Taoyuan, Taiwan; 3 Audiology and Speech Pathology Center, Chang Gung Memorial Hospital, Chiayi, Taiwan; Universidade Federal de Sao Paulo/Escola Paulista de Medicina (Unifesp/epm), BRAZIL

## Abstract

Postoperative hearing improvement is one of the main expectations for patients receiving tympanoplasty. The capacity to predict postoperative hearing may help to counsel a patient properly and avoid untoward expectations. It is difficult to predict postoperative hearing without knowing the disease process in the middle ear, which can only be assessed intraoperatively. However, the duration and extent of the underlying pathologies may represent in bone-conduction threshold and air-bone gap. Here in patients undergoing tympanoplasty without ossiculoplasty, we sorted and separated the surgery dates into the first group to build the predicting models and the second group to test the predictions. There were 87 and 30 ears, respectively. No specific enrollment or exclusion criteria were based on underlying pathologies such as the perforation size of the tympanic membrane or the middle ear conditions. The results show that bone-conduction threshold and air-bone gap together predicted air-conduction threshold after the surgery, including each frequency of 0.5k, 1k, 2k, and 4k Hz. The discrepancies between the predictions and recordings did not differ among these four frequencies. Of the variance in mean postoperative air-conduction threshold, 56.7% was linearly accounted for by these two preoperative predictors in this sample. The results suggest a trend that, the higher the frequency, the larger the part was accounted for by these two preoperative predictors. These together may help a surgeon to estimate frequency-specific hearing outcome after the surgery, answer patients’ questions with quantitative statistics, and counsel patients with proper expectations.

## Introduction

Impaired hearing is one of the main presentations in patients with chronic otitis media (COM) [1, 2], which is the main indication of tympanoplasty. Improving hearing is one goal of tympanoplasty [3–5], in addition to removing the underlying disease [5] and producing a safe dry ear [5]. It would be helpful if ear surgeons can predict the outcome of postoperative hearing and give proper counseling for the patient, to avoid untoward expectations [6].

The capacity of predicting postoperative hearing may avoid indecent expectations and enable ear surgeons to estimate other acoustic or psychometric benefits before a tympanoplasty. The magnitude of improvement in the air-conduction threshold after the surgery reportedly plays a more important role on psychometric benefit than the achievement of a certain threshold level [7].

However, it is difficult to predict postoperative hearing outcome without knowing the disease process in the middle ear, which can only be assessed intraoperatively [5]. Studies usually reported only qualitative hearing comparisons, e.g., the better outcome in two groups [6] or improvement vs. deterioration after the surgery [8]. Higashimachi et al. built a predicting model comparing the frequency response vs. stapes-footplate displacement between COM patients and normal-hearing individuals [9], without an associated report predicting real hearing outcome. Szaleniec et al. trained artificial neural network and *k*-nearest neighbor models to predict whether tympanoplasty improves or deteriorates postoperative hearing (i.e., two categorical outputs) by 21 independent variables, including age, gender, audiometric results, ear pathology, and surgical procedures [8]. In the literature, it is difficult to find a report quantitatively predicting frequency-specific air-conduction threshold after type-1 tympanoplasty.

Frequency-specific air-conduction thresholds are important [10] to patients’ postoperative hearing perception. Studies had shown that hearing before surgery (such as bone-conduction threshold and air-bone gap [8]) is associated with hearing after surgery, regardless of anatomy (e.g., see [3]). Underlying pathologies can represent in bone conduction threshold [11–15], such as infection of the middle ear apparatus [11, 12], size of perforations [12, 15, 16], and frequency of otorrhea [12, 16]. The proposed mechanisms included absorption of toxins into the perilymph [17] due to permeability changes [18] of the round window membrane, which may be associated with the extent and duration of pathologic change in the middle ear [12–14]. It is unclear what audiometric results and how they associate with postoperative hearing.

Here, we separated patients receiving tympanoplasty without ossiculoplasty into two groups according to the surgery date. Preoperative audiometric results were examined to see if there is any significant difference between these two groups. We built frequency-specific multiple-linear-regression (MLR) models for postoperative air-conduction threshold from the first (earlier) group, with 2 preoperative predictors of bone-conduction threshold and air-bone gap, and examined the statistical significance of the regressions. Predictions of the postoperative air-conduction threshold were generated according to the MLR models. Then we scatter plotted and compared the individual recorded and predicted postoperative air-conduction threshold in the second (later) group. It showed no significant difference between recorded and predicted thresholds. An analysis was then taken to show if predicted-recorded differences were similar across frequencies.

## Materials and methods

From Jul. 2013 to Oct. 2020, we enrolled patients with these criteria:

- Receiving type-1 tympanoplasty
- Having air and bone conduction pure-tone thresholds at 0.5k, 1k, 2k, and 4k Hz

Huang performed each tympanoplasty under general anesthesia. After denuding the perforation, he developed a tympanomeatal flap and placed the grafting material under the drum and the tympanomeatal flap (underlay technique). The exclusion criteria were:

- Undergoing mastoidectomy or ossiculoplasty, or
- Showing ambiguous hearing threshold, including “>100 dB”

We sorted the list with the surgery date, then preserved the last 30 surgeries as the second group to test the predictions. The rest of the earlier surgeries made the first group to build the models. The two groups had different patients with no overlapping. There were no specific enrollment or exclusion criteria based on underlying pathologies such as the perforation size of the tympanic membrane or the middle ear conditions. We conducted a t-test for preoperative air-conduction, bone-conduction thresholds, and air-bone gap between the two groups to see if there is a difference.

To know the best way to combine the two predictors and how well the predictors explain the postoperative air-conduction threshold, we scatter plotted the distribution and then developed MLR models with the following:

$$\text{Y}=\beta_{0}+\beta_{1}*{\text{X}}_{1}+\beta_{2}*{\text{X}}_{2}$$

where Y = postoperative air-conduction threshold, X_1_ = preoperative bone-conduction threshold, and X_2_ = preoperative air-bone gap. β_0_, β_1_, and β_2_ are the regression coefficients. We ran a statistic test for each MLR to see whether these two preoperative factors together predict postoperative air-conduction threshold.

Based on these models and the two preoperative thresholds of the second group, we calculated to predict the postoperative air-conduction thresholds. Then we scatter plotted individual predicated and recorded values and tested whether there is a statistical difference by a paired t-test.

We performed a one-way analysis of variance (ANOVA) on predicted-recorded difference as a function of frequency to see if predicted-recorded difference were similar across frequencies. There were 4 levels of frequencies (0.5k, 1k, 2k, and 4k Hz). The statistical significance was all tested as α = 0.05.

All statistical examinations were performed in MATLAB 9.4.0.813654 with Statistics and Machine Learning Toolbox (MathWorks, Natick, MA, USA).

### Ethical statements

The Institutional Review Board (IRB) of Chang Gung Medical Foundation approved this study (202001856B0) on Nov. 3, 2020. The IRB approved the waiver of the participants’ consent for this retrospective study. We accessed the non-anonymized medical records from Chang Gung Memorial Hospital, Chiayi, in Nov. 2020.

## Results

Between Jul. 2013 and Oct. 2020, there were 117 ears meeting the inclusion criteria. The last 30 ears in 29 patients formed the second group to test the predictions. The rest of 87 ears in 84 patients made the first group. In the first group, there were 33 men and 51 women with a mean age of 55.5 ± 17.1 (one standard deviation (SD), the same as following) years. The surgery dates ranged from Aug. 2013 to Apr. 2019. Fifty-one were right ears and 36 were left. In the second group, there were 10 men and 19 women with a mean age of 52.6 ± 17.6 years. Their surgery dates ranged from May. 2019 to Jul. 2020. There were 14 left and 16 right ears. The preoperative mean air-conduction threshold and SD were 49.5 ± 18.1 dB in group 1 and 43.3 ± 19.8 dB in group 2, t(29) = -1.73, p = 0.0946. The preoperative mean bone-conduction threshold and SD were 28.3 ± 16.0 dB in group 1 and 24.3 ± 16.8 dB in group 2, t(29) = -1.31, p = 0.2017. The preoperative mean air-bone gap and SD were 21.2 ± 10.4 dB in group 1 and 19.0 ± 10.4 dB in group 2, t(29) = -1.17, p = 0.2506. There was no significant preoperative hearing difference in these two groups. The postoperative hearing was measured 73 ± 48.7 days after the surgery in group 1 and 74 ± 49.1 days in group 2.

**Fig 1:**
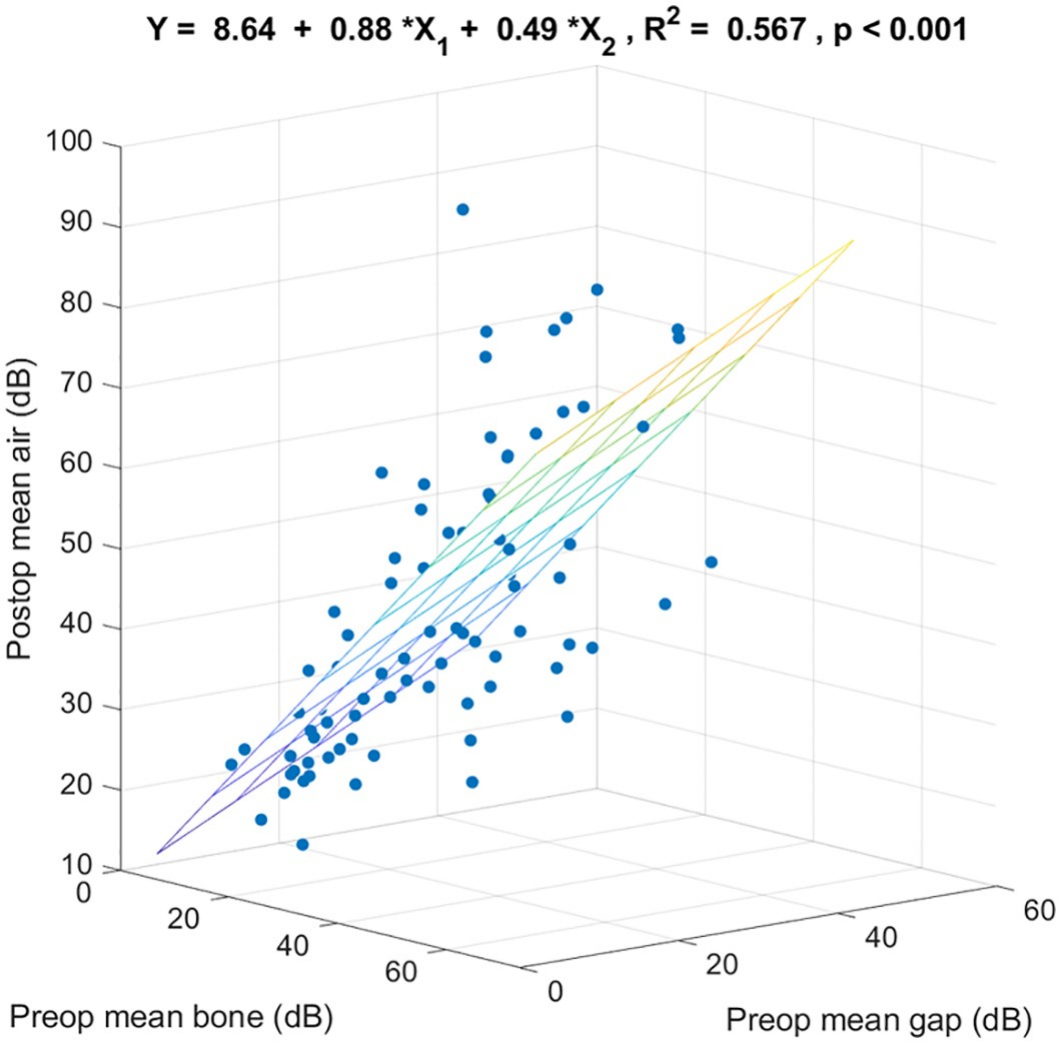


We built the regression models from the first group. Fig 1 shows the 3-dimensional scatter plot and the regression coefficients specified by mean preoperative bone-conduction threshold, mean preoperative air-bone gap, and mean postoperative air-conduction threshold of 0.5k, 1k, 2k, and 4k Hz. These 2 preoperative variables together significantly predicted mean postoperative air-conduction thresholds (R^2^ = 0.567, p < 0.001). Of the variance in mean postoperative air-conduction threshold, 56.7% was linearly accounted for by mean preoperative bone-conduction threshold and mean preoperative air-bone gap in this sample. For any mean preoperative air-bone gap, on the average each additional dB of mean preoperative bone-conduction threshold was associated with an increase in mean postoperative air-conduction threshold of 0.88 dB. For any mean preoperative bone-conduction threshold, on the average each additional dB of mean preoperative air-bone gap was associated with an increase in mean postoperative air-conduction threshold of 0.49 dB.

**Fig 2:**
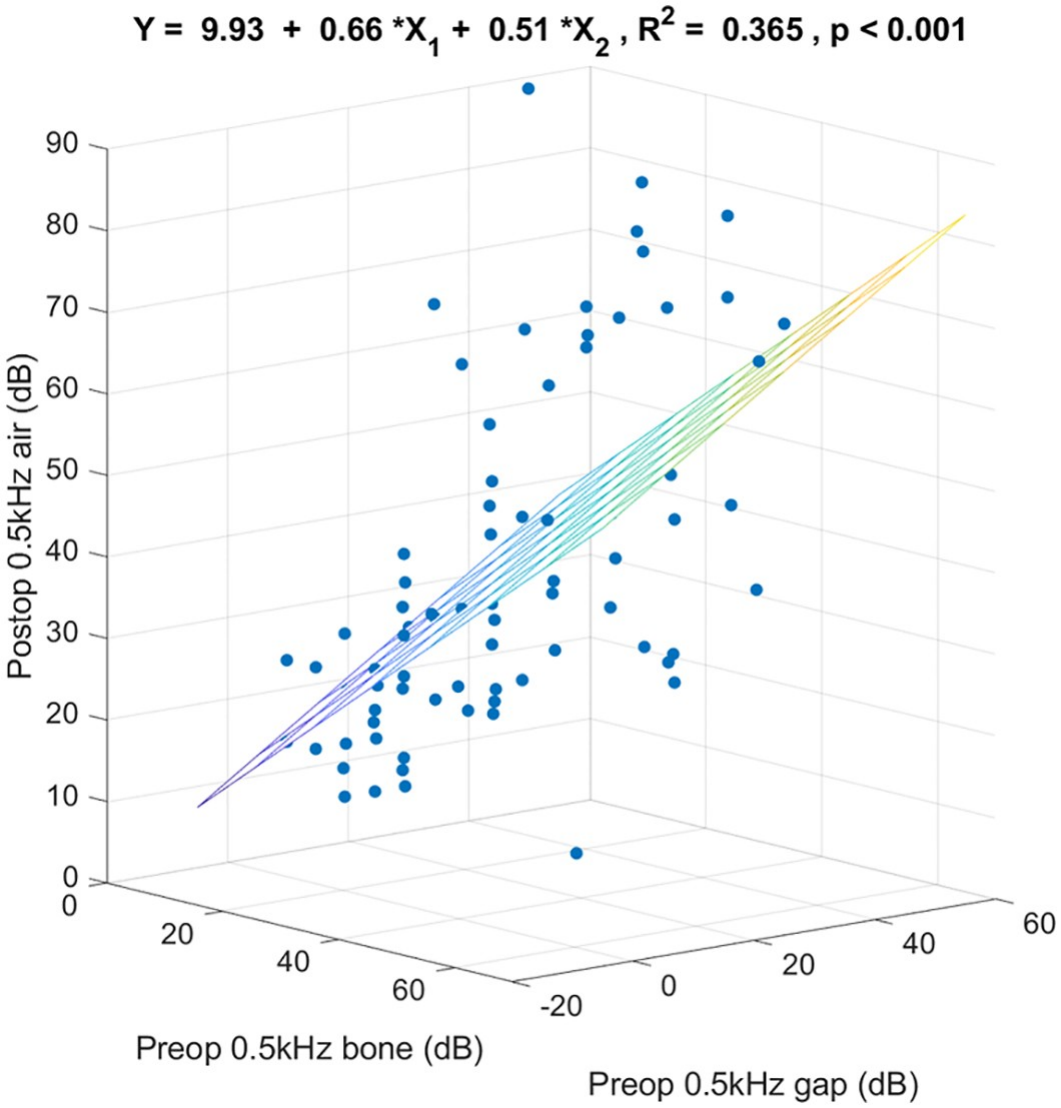


Fig 2 shows the 3-dimensional scatter plot and the regression coefficients specified by preoperative 0.5k Hz bone-conduction threshold, preoperative 0.5k Hz air-bone gap, and postoperative 0.5k Hz air-conduction threshold. These 2 preoperative variables together predicted postoperative 0.5k Hz air-conduction thresholds (R^2^ = 0.365, p < 0.001). Of the variance in postoperative 0.5k Hz air-conduction threshold, 36.5% was linearly accounted for by preoperative 0.5k Hz bone-conduction threshold and preoperative 0.5k Hz air-bone gap in this sample. For any given preoperative 0.5k Hz air-bone gap, on the average each additional dB of preoperative 0.5k Hz bone-conduction threshold was associated with an increase in postoperative 0.5k Hz air-conduction threshold of 0.66 dB. For any given preoperative 0.5k Hz bone-conduction threshold, on the average each additional dB of preoperative 0.5k Hz air-bone gap was associated with an increase in postoperative 0.5k Hz air-conduction threshold of 0.51 dB.

**Fig 3:**
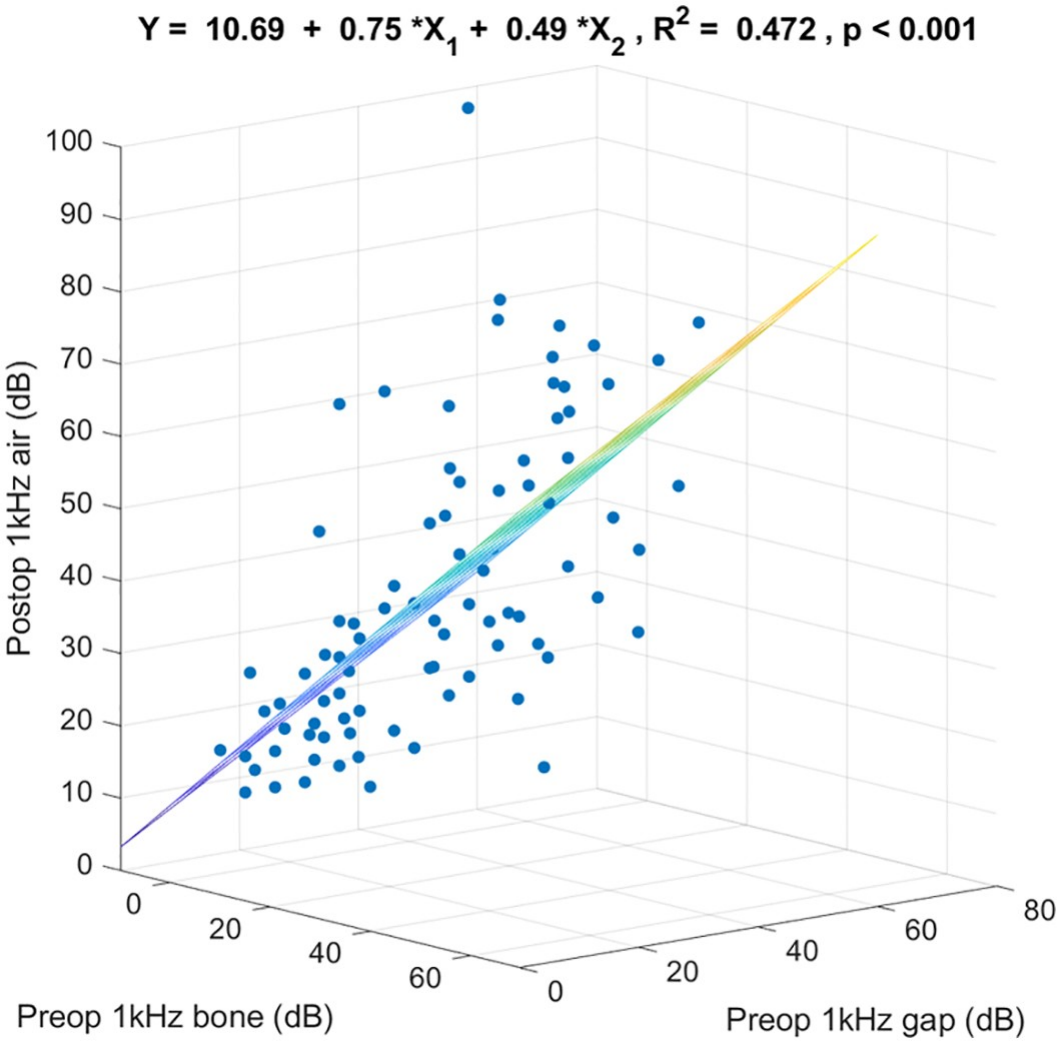


Fig 3 shows the 3-dimensional scatter plot and the regression coefficients specified by preoperative 1k Hz bone-conduction threshold, preoperative 1k Hz air-bone gap, and postoperative 1k Hz air-conduction threshold. These 2 preoperative variables together predicted postoperative 1k Hz air-conduction thresholds (R^2^ = 0.472, p < 0.001). Of the variance in postoperative 1k Hz air-conduction threshold, 47.2% was linearly accounted for by preoperative 1k Hz bone-conduction threshold and preoperative 1k Hz air-bone gap in this sample. For any given preoperative 1k Hz air-bone gap, on the average each additional dB of preoperative 1k Hz bone-conduction threshold was associated with an increase in postoperative 1k Hz air-conduction threshold of 0.75 dB. For any given preoperative 1k Hz bone-conduction threshold, on the average each additional dB of preoperative 1k Hz air-bone gap was associated with an increase in postoperative 1k Hz air-conduction threshold of 0.49 dB.

**Fig 4:**
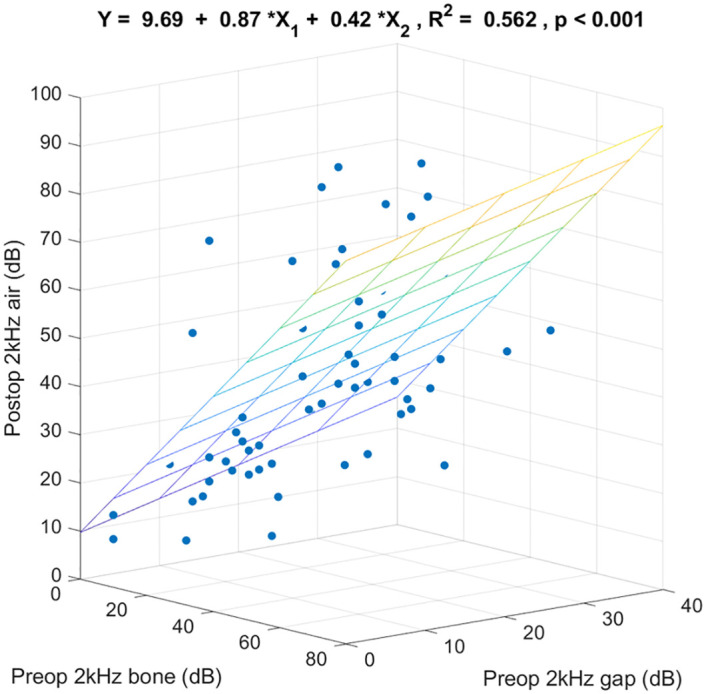


Fig 4 shows the 3-dimensional scatter plot and the regression coefficients specified by preoperative 2k Hz bone-conduction threshold, preoperative 2k Hz air-bone gap, and postoperative 2k Hz air-conduction threshold. These 2 preoperative variables together predicted postoperative 2k Hz air-conduction thresholds (R^2^ = 0.562, p < 0.001). Of the variance in postoperative 2k Hz air-conduction threshold, 56.2% was linearly accounted for by preoperative 2k Hz bone-conduction threshold and preoperative 2k Hz air-bone gap in this sample. For any given preoperative 2k Hz air-bone gap, on the average each additional dB of preoperative 2k Hz bone-conduction threshold was associated with an increase in postoperative 2k Hz air-conduction threshold of 0.87 dB. For any given preoperative 2k Hz bone-conduction threshold, on the average each additional dB of preoperative 2k Hz air-bone gap was associated with an increase in postoperative 2k Hz air-conduction threshold of 0.42 dB.

**Fig 5:**
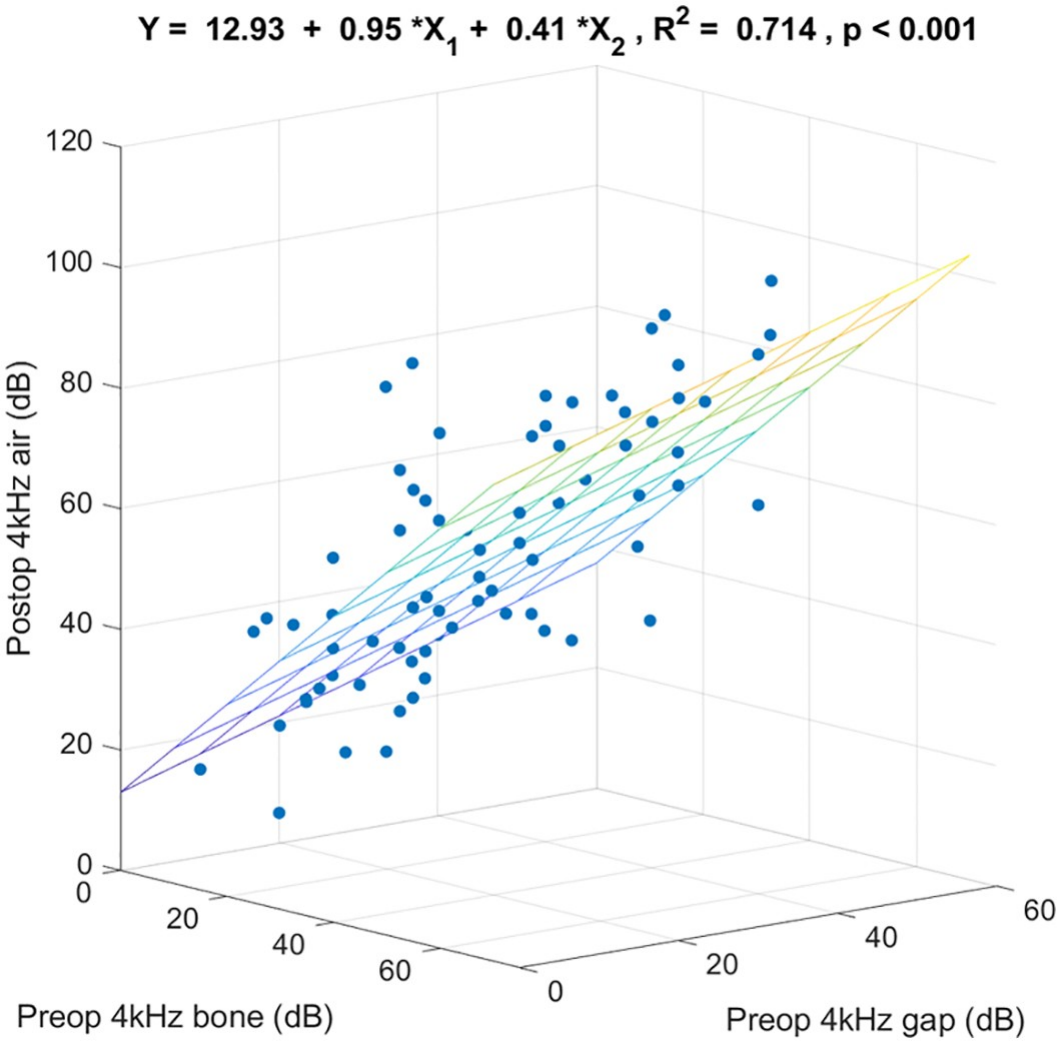


Fig 5 shows the 3-dimensional scatter plot and the regression coefficients specified by preoperative 4k Hz bone-conduction threshold, preoperative 4k Hz air-bone gap, and postoperative 4k Hz air-conduction threshold. These 2 preoperative variables together predicted postoperative 4k Hz air-conduction thresholds (R^2^ = 0.714, p < 0.001). Of the variance in postoperative 4k Hz air-conduction threshold, 71.4% was linearly accounted for by preoperative 4k Hz bone-conduction threshold and preoperative 4k Hz air-bone gap in this sample. For any given preoperative 4k Hz air-bone gap, on the average each additional dB of preoperative 4k Hz bone-conduction threshold was associated with an increase in postoperative 4k Hz air-conduction threshold of 0.95 dB. For any given preoperative 4k Hz bone-conduction threshold, on the average each additional dB of preoperative 4k Hz air-bone gap was associated with an increase in postoperative 4k Hz air-conduction threshold of 0.41 dB.

**Fig 6:**
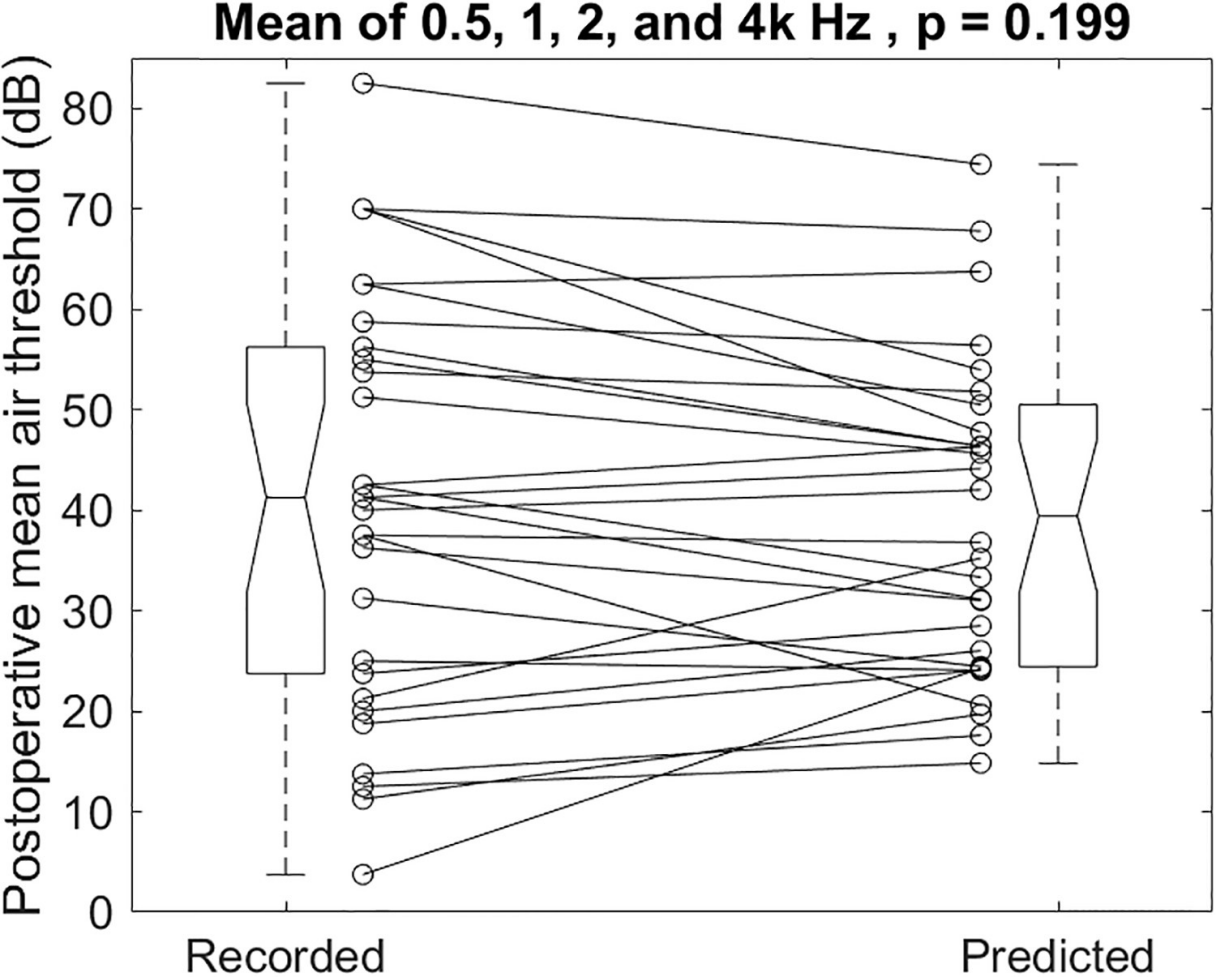


In the second group, we verified the predictions by scatter plotting the recorded postoperative mean air-conduction thresholds on the left side in Fig 6 and those predicted by the two preoperative thresholds on the right. The individual recorded and predicted results illustrated in Fig 6 had a mean and SD of 41.5 ± 20.5 and 39.4 ± 15.6 dB, respectively (p = 0.199). The recorded postoperative mean air-conduction thresholds were not different from the predicted ones.

**Fig 7:**
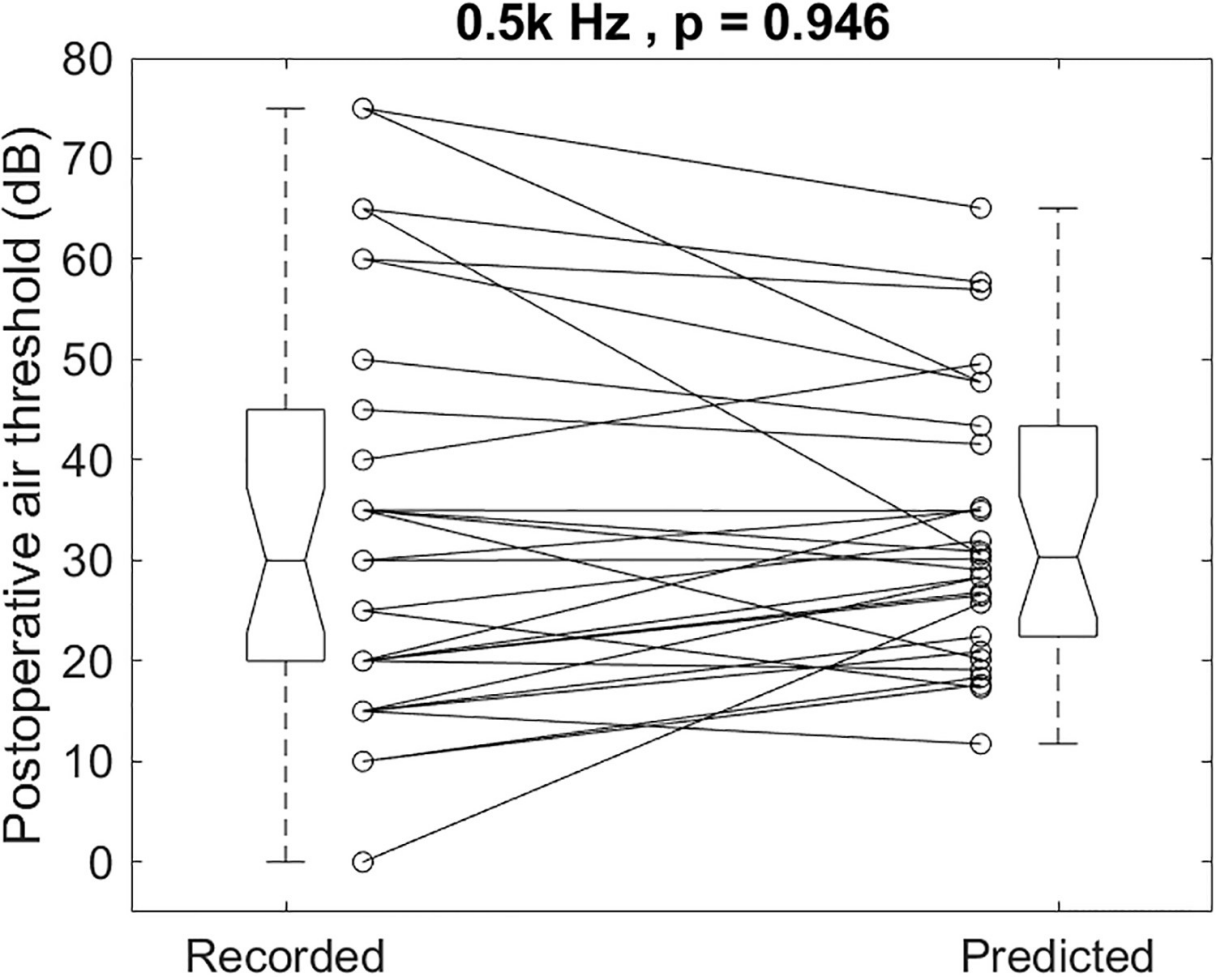


The individual recorded and predicted results illustrated in Fig 7 for 0.5k Hz had a mean and SD of 33.5 ± 20.3 and 33.3 ± 13.6 dB, respectively (p = 0.946). The recorded postoperative 0.5k Hz air-conduction thresholds were not different from the predicted ones.

**Fig 8:**
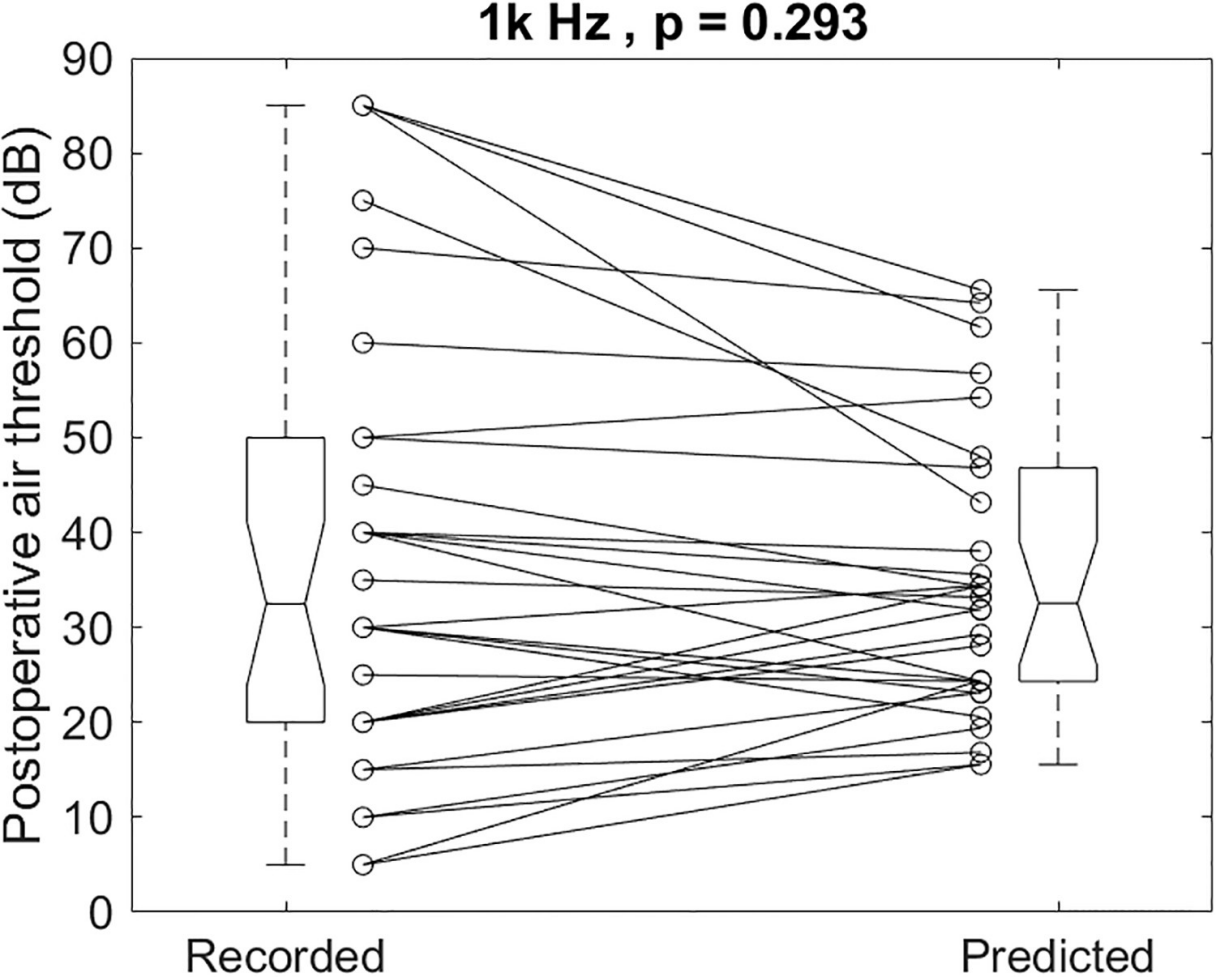


The individual recorded and predicted results illustrated in Fig 8 for 1k Hz had a mean and SD of 37.8 ± 23.9 and 35.2 ± 14.9dB, respectively (p = 0.293). The recorded postoperative 1k Hz air-conduction thresholds were not different from the predicted ones.

**Fig 9:**
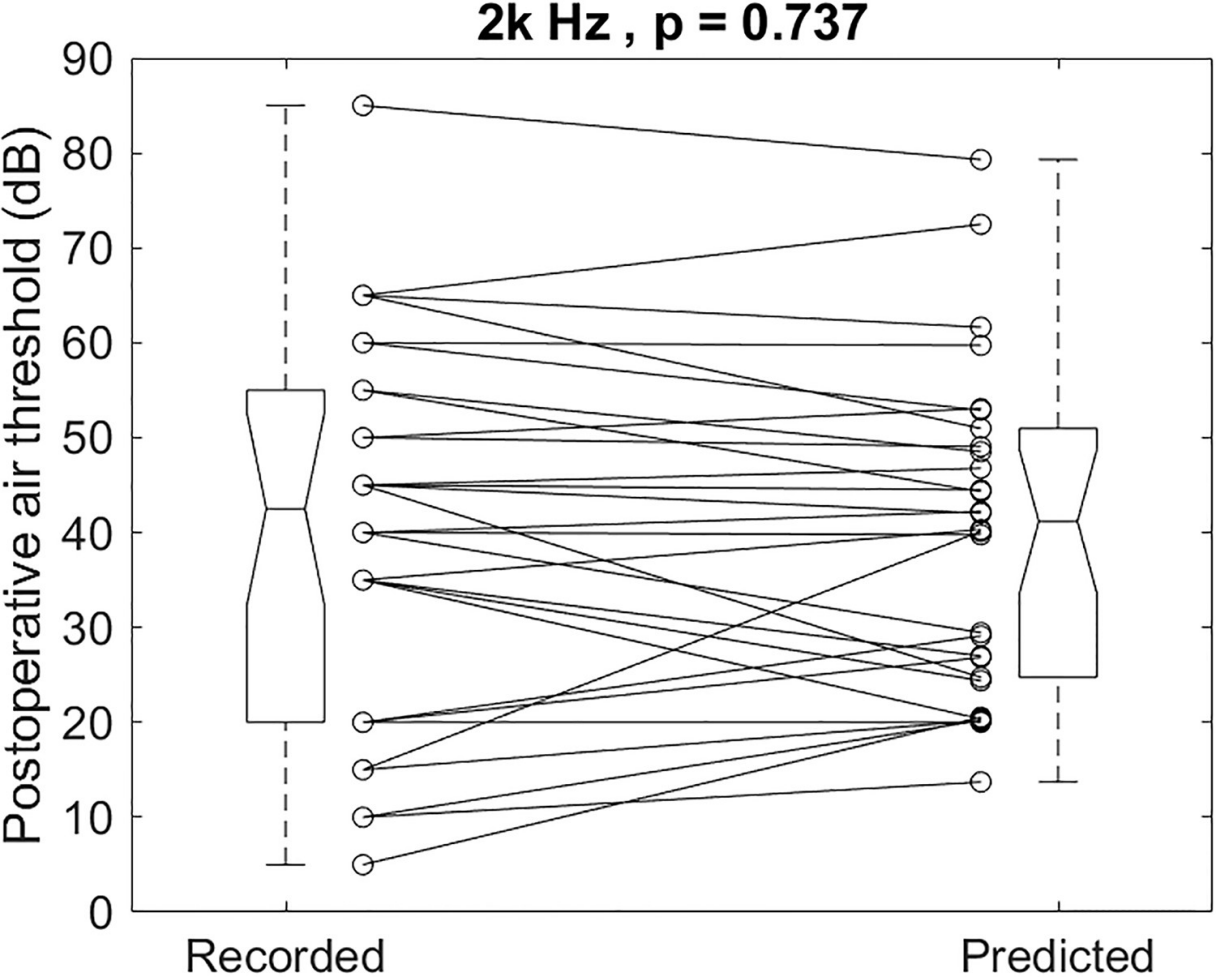


The individual recorded and predicted results illustrated in Fig 9 for 2k Hz had a mean and SD of 40.5 ± 19.6 and 39.9 ± 16.6dB, respectively (p = 0.737). The recorded postoperative 2k Hz air-conduction thresholds were not different from the predicted ones.

**Fig 10:**
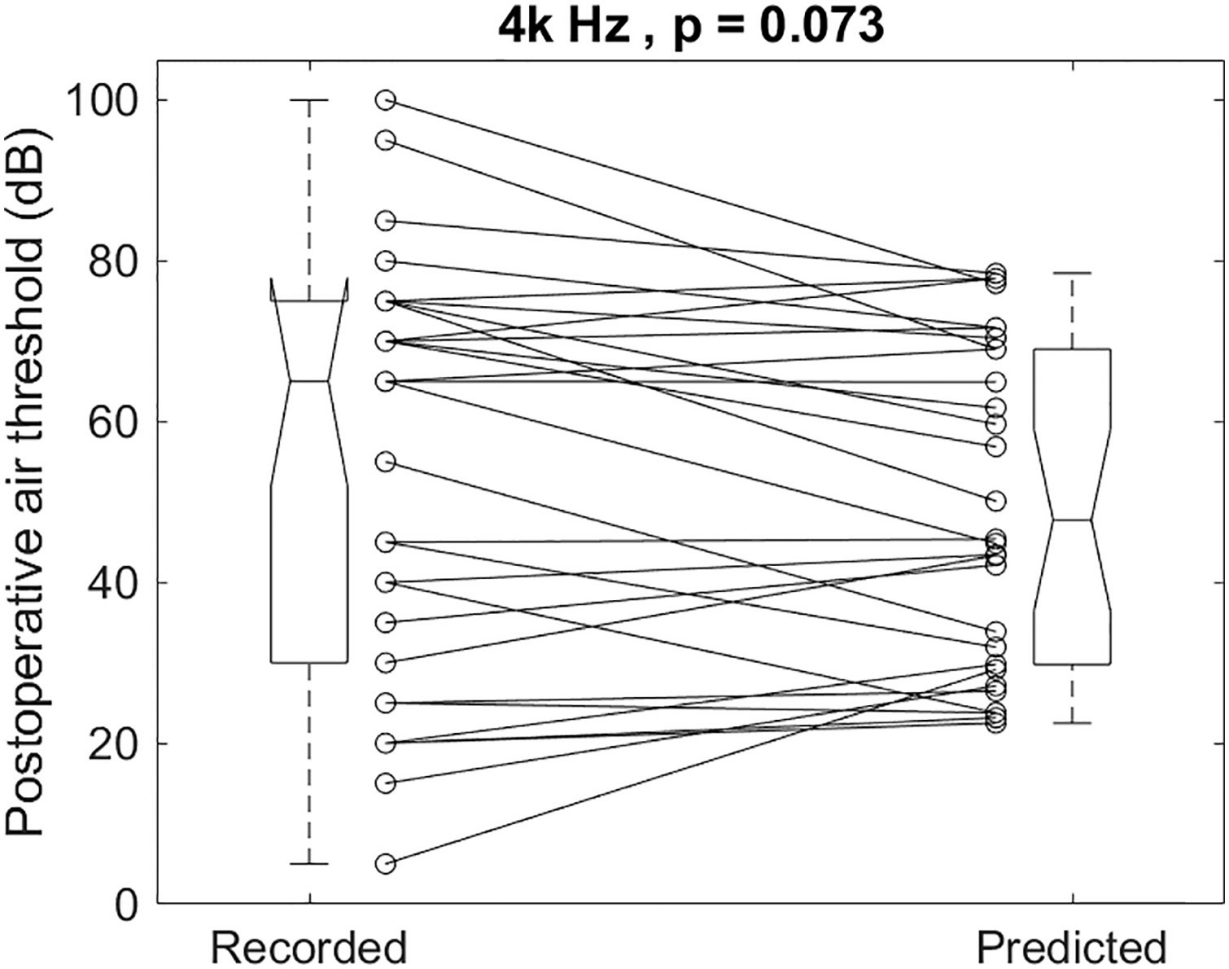


The individual recorded and predicted results illustrated in Fig 10 for 4k Hz had a mean and SD of 54.3 ± 26.0 and 49.9 ± 19.7dB, respectively (p = 0.073). The recorded postoperative 4k Hz air-conduction thresholds were not different from the predicted ones.

**Fig 11:**
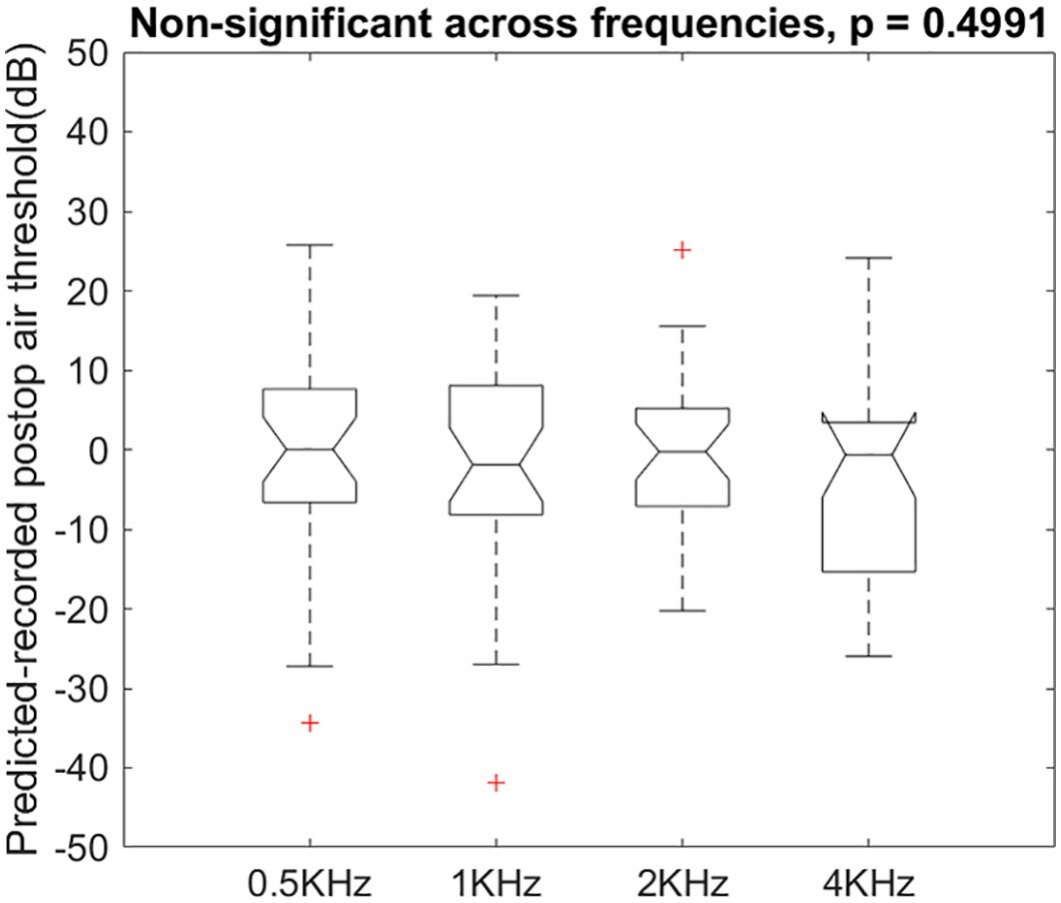


The ANOVA test showed there was no difference on (predicted—recorded) thresholds among the levels of frequencies, F(3, 116) = 0.79, p = 0.499 (Fig 11).

## Discussion

Researchers pursuing a successful tympanoplasty have been looking for predictors for an optimal postoperative hearing. There seemed to be no universally accepted predictor. Numerous variables had been studied, including age [8, 19], audiometric results [8], absence of craniofacial dysmorphia [19], state of contralateral ear [19], state of otorrhea and infection [20, 21], previous adenoidectomy [19], cause of perforation [19], size of perforation [8, 19], state of middle-ear mucosa [8, 19, 20], residual ossicular remnants [22–24], allergy [25–28], and Eustachian tube function [6, 20, 25]. In the available literature, it is difficult to find a report quantitatively predicting frequency-specific air-conduction threshold after tympanoplasty. However, these variables or underlying pathologies may partially present in the preoperative bone-conduction threshold and air-bone gap [11–14, 16–18].

Our results show that bone-conduction threshold and air-bone gap could quantitatively predict frequency-specific air-conduction threshold after tympanoplasty. Of the variance in postoperative mean air-conduction threshold, more than half (56.7%) was linearly accounted for by preoperative mean bone-conduction threshold and preoperative mean air-bone gap in this sample. The higher the frequency, the larger the part was accounted for by these 2 preoperative predictors. This could be related to the findings of worse bone-conduction thresholds at higher frequencies [16]. The discrepancies between the predictions and recordings did not differ among 0.5k, 1k, 2k, and 4k Hz. The magnitude of postoperative improvement in the air-conduction threshold plays an important role on psychometric benefit [7]. The results may help a surgeon to avoid indecent expectations and to estimate psychometric benefits before the surgery. In the present study, we did not try to predict speech discrimination, which had been reported not to be influenced by disease [12]. The sample consists of patients treated by a single surgeon, limiting generalizability of the results. It needs future studies to analyze speech discrimination and whether data from other surgeons (e.g., within one or between two surgeons) will reproduce similar findings and trends.

The results show that 56.7% of the variance in postoperative mean air-conduction threshold was linearly accounted for by preoperative mean bone-conduction threshold and preoperative mean air-bone gap in this sample. This suggests there were unknown variables accounting for a little less than half of the variance, and these unknown variables were more important at lower frequencies. Singer et al. reported that bone-conduction threshold may be associated with predisposing factors such as diabetes mellitus and smoking [16]. It needs future studies to discover what these variables are and why they affect more at low frequencies.

## Conclusions

Impaired hearing is one of the main presentations and concerns in COM patients. Being able to predict postoperative hearing may avoid indecent expectations and help ear surgeons to estimate other acoustic or psychometric benefits before a tympanoplasty. The results show that, of the variance in postoperative mean air-conduction threshold, more than half (56.7% in this sample) was linearly accounted for by these two predictors, which may reflect the duration and extent of the underlying pathologies. There seems to be a trend that, the higher the frequency, the larger the part was accounted for by these 2 predictors.
